# Comparison of different methods for reducing pain during a heel lance in newborns: a randomized trial

**DOI:** 10.1186/s13052-025-01916-w

**Published:** 2025-03-12

**Authors:** Tuğba Karga Yilmaz, Betul Yavuz

**Affiliations:** 1 Uşak Training and Research Hospital, Uşak, Türkiye; 2 Faculty of Health Sciences, Department of Pediatric Nursing, Kütahya Health Sciences University, Kütahya, Türkiye

**Keywords:** Breastfeeding, Pain, SSC, Swaddling + holding

## Abstract

**Background:**

This study aimed to compare three different methods [breastfeeding, skin-to-skin contact (SSC), swaddling + holding] to reduce the pain felt by term newborns during a heel lance (HL).

**Methods:**

This was a randomized three-group experimental study. The study sample included 90 newborns, 30 in each group. The data were collected using a pulse oximeter, a voice recorder, an Information Form, the Neonatal Infant Pain Scale (NIPS). The newborns’ pain level, heart rate, and oxygen saturation were measured at three different times.

**Results:**

No difference was found between the groups’ mean NIPS scores before the HL procedure (*p* > 0.05). The mean NIPS scores 10 s after the procedure started and after the HL procedure were the lowest in the breastfeeding group, followed by the SSC and swaddling + holding groups (*p* < 0.05). There was no difference between the groups’ mean heart rates before the procedure; however, there was a significant difference 10 s after the procedure started and after the procedure (*p* < 0.05).

**Conclusion:**

Breastfeeding is the most effective method to reduce pain during and after HL procedures in term newborns, followed by SSC and swaddling + holding.

**Trial registration:**

This study was retrospectively registered at ClinicalTrials.gov with the registration number NCT05797532 (date: 04.04.2023).

## Introduction

Newborns are exposed to painful invasive procedures from the first hours of their lives [1, 2]. Invasive procedures such as intravenous blood collection, heel lance (HL), venous or arterial catheterization, and subcutaneous and intramuscular injections conducted for diagnostic and therapeutic purposes cause pain perception in newborns [1] and thus increase heart rate [3–8] and crying [3–5, 7, 9–12], decrease oxygen saturation [3]. These painful invasive procedures, when inadequately treated, can lead to long-term adverse effects (altered brain development, dysregulation of the hypothalamic-pituitary-adrenal axis) in neonates exposed to repetitive pain and stress. The detrimental impact of cumulative procedural pain on neonatal brain development highlights the importance of recognizing and effectively managing pain in neonates [13].

HL for metabolic screening of newborns is one of the painful invasive procedures [1]. The collection of capillary blood for a screening test by a puncture of the medial lateral side of the heel is associated with pain experienced by newborns. This technique is popular because it enables the collection of a very small volume of blood (0.2–0.5 mL). The use of this technique requires proper preparation of the child’s foot, adherence to the principles of infection control measures before and during the procedure, and the use of non-pharmacological methods of pain relief before starting the procedure [14].

There are studies examining the effect of nonpharmacologic methods in reducing the severity of pain felt by newborns during the HL, for example, breast milk [15], swaddling [3, 9], holding [6, 9, 11], breastfeeding [10, 11, 16–20], music [20, 21], oral sucrose [15, 17, 22], non-nutritive sucking [17, 21], skin-to-skin contact (SSC) [5, 10, 17], SSC + breastfeeding [7, 22], SSC + sucrose [22], swaddling + holding, swaddling + holding + breastfeeding [12], breastfeeding + holding [6], kangaroo care [4, 8] and positioning [18, 23]. SSC, breastfeeding, and swaddling + holding are methods, which are natural, economical, easily accessible, do not require preparation, and are effective in maintaining mother-newborn bonding. They can be easily used by mothers and nurses to reduce pain during the HL.

The newborn screening program is the only procedure that enables early detection, diagnosis and treatment of several dozen congenital diseases that are life-threatening, disturb development and lead to irreversible neurological changes and severe intellectual disability [14]. In this context, nurses who routinely perform HLs for metabolic screening of newborns in maternity wards of hospitals and Family Health Centers have a key role in reducing the pain level of newborns.

According to the American Academy of Pediatrics (AAP), pain reduction in newborns remains an area that needs to be better addressed. However, new data should be added to the body of knowledge regarding pain management among neonates [24]. This study was conducted to compare the efficacy of three different methods (breastfeeding, SSC, and swaddling + holding) in reducing the pain felt by term newborns during the HL.

## Materials and methods

### Study design

This was a randomized, three-group experimental study. The population of this study consisted of all newborns born in a training and research hospital in western Turkey. The sample of the study consisted of 90 newborns born in the obstetrics department of this hospital between 18 November 2019 and 31 May 2020, who met the inclusion criteria.

### Population and participants

**Fig. 1:**
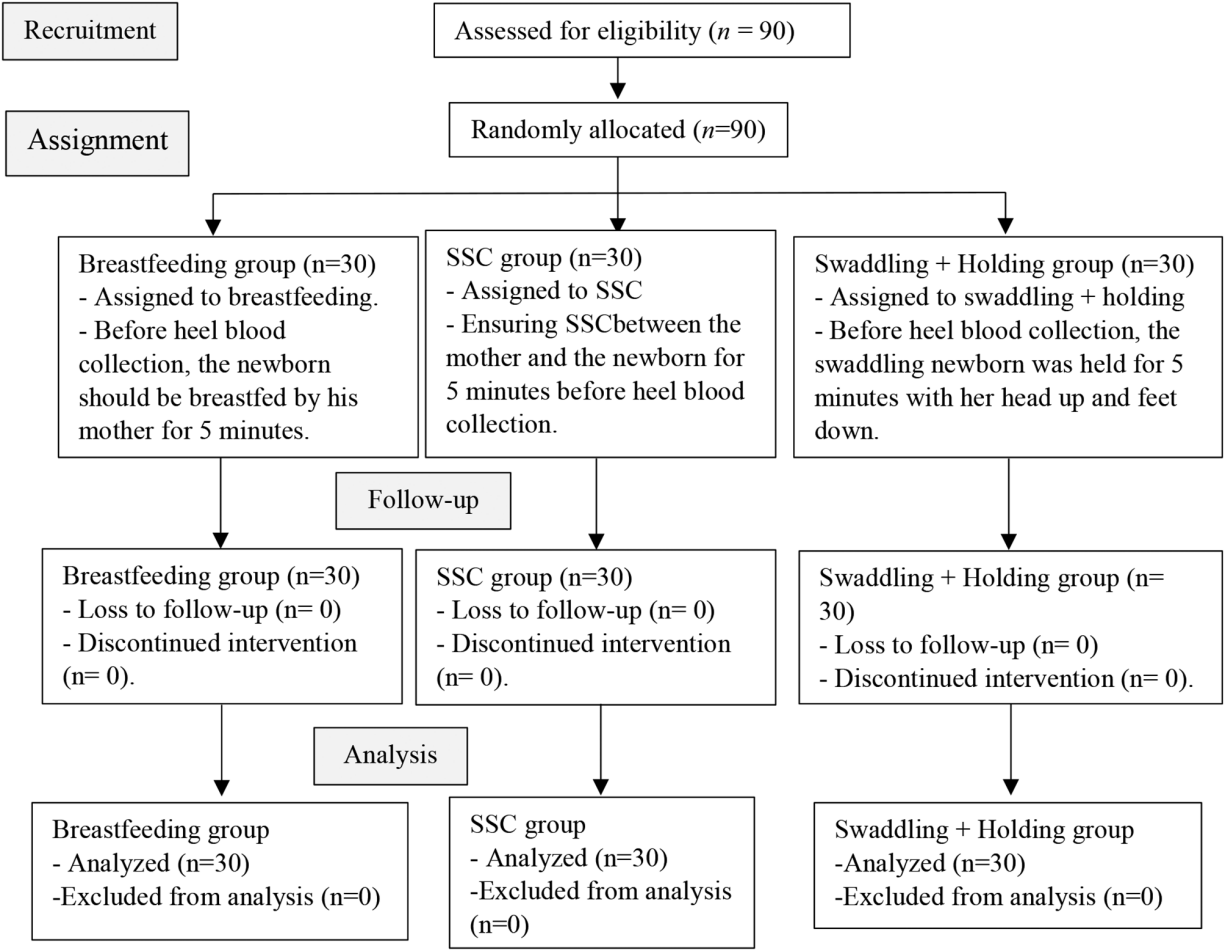
Participant selection flowchart

The sample group should include at least 30 participants for parametric measurements and experimental studies [25]. Therefore, the sample of the study consisted of a total of 90 newborns, 30 in each study group (Fig. 1).

The inclusion criteria were as follows: (1) Newborns (0–28 days) who were born at term (38–42 weeks), (2) had stable vital signs, (3) were at least 24 h old, (4) were fed, (5) received the HL for routine metabolic screening, (6) did not undergo any other invasive intervention except for a vitamin K injection and the hepatitis B vaccine, (7) had an Apgar score ≥ 7 at the 1st and 5th min, and (8) whose parents gave written consent to be included in the study.

### Sample size

The eta-squared (*η*^*2*^) from a one-way repeated measures analysis of variance is an index commonly used as an effect size resulting from a post-hoc power analysis. In this study, the effect size was obtained from the results of the analysis of variance using the SAS program, since the statistic *F* can be expressed as a function of *η*^*2*^. In this context, at the end of the study, *η*^*2*^ was 0.68 and the sample power was 0.99, based on the mean NIPS score.

### Sampling and randomization

The newborns included in the study were randomly divided into three groups: SSC (*n* = 30), swaddling + holding (*n* = 30), and breastfeeding (*n* = 30).

For randomization, the names of each method (breastfeeding, skin-to-skin contact, and swaddling + holding) were written on different pieces of paper by the researcher. These papers were put into envelopes (90 in total) and these envelopes were sealed and put into a bag. The parents selected one of these envelopes and opened it in the presence of the researcher.

### Data collection tools

#### Parent and newborn information form

This form prepared by the researchers included questions about the socio-demographic data of the newborns (gestational week, gender, birth length, weight, Apgar scores, and mode of delivery), parents (maternal age, education level, maternal employment, and planned pregnancy) as well as a follow-up chart in which measurements (oxygen saturation and heart rate) were recorded.

#### Neonatal infant pain scale (NIPS)

The NIPS was developed by Lawrence et al. (1993) to assess pain during invasive interventions in premature babies and newborns. Akdovan (1999) adapted the NIPS into Turkish [26]. NIPS is a scale that evaluates acute procedural pain in newborns [13]. The scale includes five behavioral parameters (facial expression, crying, arm movements, leg movements, and state of arousal) and one physiological parameter (breathing patterns). Each behavior is scored 0–1; the crying parameter is scored 0–1–2. The lowest score is 0 and the highest score is 7. Cronbach’s alpha coefficients of the scale were 0.95, 0.87, and 0.88 before, during, and after the procedure, respectively [27].

#### Pulse oximeter and saturation probe

A Freely SHO 6002 manufactured in Shanghai, China, calibrated pulse oximeter device, and saturation probe were attached to the newborn’s foot (left) that was not receiving any procedures, and oxygen saturation and heart rate were measured.

#### Stopwatch

The stopwatch of an Apple iPhone SE mobile phone was used to measure the crying time of newborns during the HL and the duration of the procedure.

#### Voice recorder

Crying sound was recorded using an Apple iPhone SE mobile phone to determine the crying time of newborns during the procedure. The voice recorder was turned on 2 min before the HL and off 2 min after the procedure.

#### Needle

A 22 G (0.7-x50 mm-black) needle was used for the HL.

### Intervention protocols

Data were collected by the researcher in the gynecology and obstetrics service of a training and research hospital between 18 November 2019 and 31 May 2020.

#### Stage 1

Before the HL procedure, parents were informed about the purpose of the study. The data were collected with written consent from the parents. The parents’ information was obtained through face-to-face interviews and the newborns’ information was obtained from their medical files.

#### Stage 2

Before the HL procedure, the probe of the pulse oximetry device was attached to the left foot of the newborn. HL procedures for all newborns were performed by a nurse. The nurse inserted a 22 G needle into the outer side of the right heel of the newborn and the heel blood was dripped onto five separate circles on Guthrie filter paper.

##### Breastfeeding group

The mother was allowed to sit comfortably on her bed in the ward. After the pulse oximeter probe was attached to the left foot of the newborn, it was placed on the mother’s lap. The newborn was allowed to breastfeed for 5 min before the HL procedure and breastfeeding was continued during the procedure.

##### SSC group

After the mother was given a 45–60-degree semi-fowler position on her bed in the ward, she was allowed to remove her upper nightgown and covered with a blanket. Then, the newborn’s clothes were taken off by the researcher, leaving only the diaper and a baby hat, and a pulse oximeter probe was connected to the left foot. The newborn was placed in the prone position on the mother’s bare chest between the two breasts, facing the mother’s face, with her head elevated and covered with a baby blanket. SSC was performed between the mother and the newborn for at least 5 min before the HL procedure.

##### Swaddling + holding group

Each newborn was placed on a square blanket with legs in flexion and abduction position and a pulse oximetry probe was attached to the left foot. The newborn was then wrapped with the blanket and placed in the mother’s lap. Before the HL procedure, the newborn was held in the mother’s lap for 5 min with the head up and the feet down.

The newborns’ pain level, heart rate, and oxygen saturation measurements were performed at three different times: (1) before the HL procedure, (2) 10 s after the procedure started, and (3) after the procedure (2 min after the procedur is completed). The voice recorder and stopwatch were turned on 2 min before and off 2 min after the HL procedure (total procedure time) to determine the crying times of the newborns in the study groups.

### Statistical analysis

Statistical analyses of the data obtained from the study were conducted using the SAS and IBM SPSS (Statistical Package for the Social Sciences) software package programs. Mean, standard deviation, number, percentage, minimum, and maximum values were used in the evaluation of the data. The Shapiro-Wilk’s test was used for the normality assumption of the variables and Levene’s test was used for the homogeneity assumption of variance. The chi-square test was used to assess the relationship between two variables, and Fisher’s exact test was used when the values were less than 5. The three groups were compared using one-way ANOVA in cases when the data were normally distributed and the Kruskal-Wallis H test when the data were not normally distributed. Dunn’s test with Bonferroni correction was used to determine from which group or measurement time the difference originated. The significance level was accepted as *p* < 0.05 [25].

## Results

The groups were similar to each other in terms of the mother’s education level, employment status, planned pregnancy, and mode of birth (*p* > 0.05); there was a difference in the mean age of the mother between the study groups (*p* < 0.05). In addition, the groups were similar to each other in terms of the 1st and 5th min Apgar scores, weight at birth, height at birth, gestational age, and sex of the newborns participating in the study (*p* > 0.05) (Table 1).

**Table 1:** Distribution of sociodemographic characteristics of the study groups

Variable	Breastfeeding		SSC		Swaddling+ holding		Test, *p*-value
	M ± *SD*		M ± *SD*		M ± *SD*		
Mother’s age	29.77 **±** 5.80		29.43 **±** 6.37		26.27 **±** 4.61		**0.034** ^**F**^
1st min APGAR Score	8.77 **±** 0.50		8.93 **±** 0.25		8.90 **±** 0.31		0.163 ^***X2***^
5th min APGAR Score	9.60 **±** 0.50		9.77 **±** 0.43		9.83 **±** 0.38		0.112 ^***X2***^
Weight at birth (g)	3195.00 **±** 338.73		3207.83 **±** 343.86		3288.50 **±** 256.38		0.464^F^
Height at birth(cm)	50.07 **±** 0.78		49.93 **±** 1.01		50.00 **±** 0.69		0.892 ^***X2***^
Gestational age	38.47 **±** 0.73		38.67 **±** 0.88		38.77 **±** 0.86		0.332 ^***X2***^
	***n***	**%**	***n***	**%**	***n***	**%**	**Test**, ***p-value***
**Newborn’s sex**							
Female	12	40.0	18	60.0	15	50.0	0.3012^*^
Male	18	60.0	12	40.0	15	50.0	
**Mode of birth**							
Cesarean	16	53.3	17	56.7	12	40.0	0.393^*^
Normal birth	14	46.7	13	43.3	18	60.0	
**Mother’s educational status**							
Primary school	19	63.3	15	50.0	17	56.7	0.102^**^
High school	5	16.7	9	30.0	12	40.0	
Undergraduate and higher	6	20.0	6	20.0	1	3.3	
**Mother’s employment status**							
Employed	4	13.3	7	23.3	1	3.3	0.084^**^
Unemployed	26	86.7	23	76.7	29	96.7	
**Was pregnancy planned**							
Yes	23	76.7	22	73.3	23	76.7	0.941^*^
No	7	23.3	8	26.7	7	23.3	

M: Mean, SD: Standard Deviation, SSC: skin-to-skin contact, F: ANOVA, *X*^*2*^: Kruskal-Wallis H test, *Chi-square analysis, **Fisher’s exact test

### Comparison of mean NIPS scores of the groups

No statistically significant difference was found between the study groups’ mean NIPS scores before the procedure (*p* > 0.05).

**Fig. 2:**
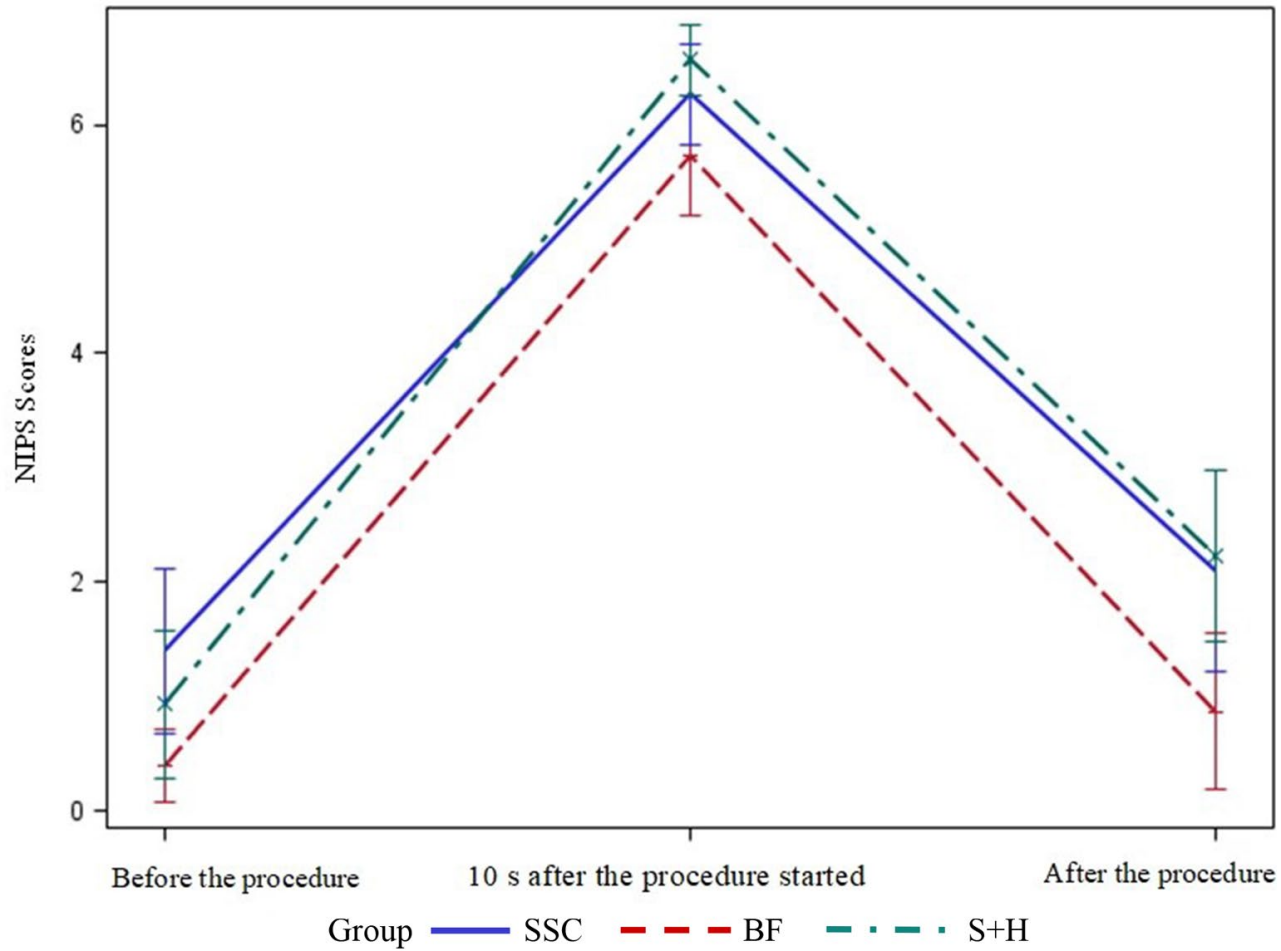
Distribution of Neonatal Infant Pain Scale (NIPS) Scores of Newborns by Group. SSC: Skin-to-Skin Contact, BF: Breastfeeding, S + H: Swaddling + Holding

There was a statistically significant difference between the study groups’ mean NIPS scores 10 s after the procedure started (*p* < 0.05) (Table 2; Fig. 2). When the groups were compared pairwise, the NIPS score of the breastfeeding group at the 10th second after the procedure started was significantly lower than that of the swaddling + holding group (*p* = 0.029).

**Table 2:** Distribution of neonatal infant pain scale (NIPS) scores of newborns by group

NIPS Scores	Experimental groups										
	Breastfeeding^a^			SSC^b^			Swaddling + holding^c^			X^2^	*p*
	M ± SD	Median	Min-Max	M ± SD	Median	Min-Max	M ± SD	Median	Min-Max		
Before the procedure	0.40 **±** 0.86	0.0	0–3	1.40 **±** 1.94	0.0	0–6	0.93 **±** 1.72	0.0	0–5	4.240	0.120
10 s after the procedure started	5.73 **±** 1.41	6.0	2–7	6.27 **±** 1.17	7.0	4–7	6.57 **±** 0.82	7.0	4–7	6.995	0.030 **a < c**
After the procedure	0.87 **±** 1.83	0.0	0–7	2.10 **±** 2.35	1.5	0–7	2.23 **±** 2.01	2.0	0–6	10.585	0.005 **a < c**,** b**

M: Mean, SD: Standard Deviation, SSC: skin-to-skin contact, *X*^*2*^: Kruskal-Wallis Test

A statistically significant difference was found between the study groups’ mean NIPS scores after the HL procedure (*p* < 0.05) (Table 2; Fig. 2). When the groups were compared pairwise, the breastfeeding group’s mean NIPS score after the procedure was significantly lower than that of the SSC group (*p* = 0.031) and the swaddling + holding group (*p* = 0.008).

### Comparison of mean heart rate scores of the groups

**Fig. 3:**
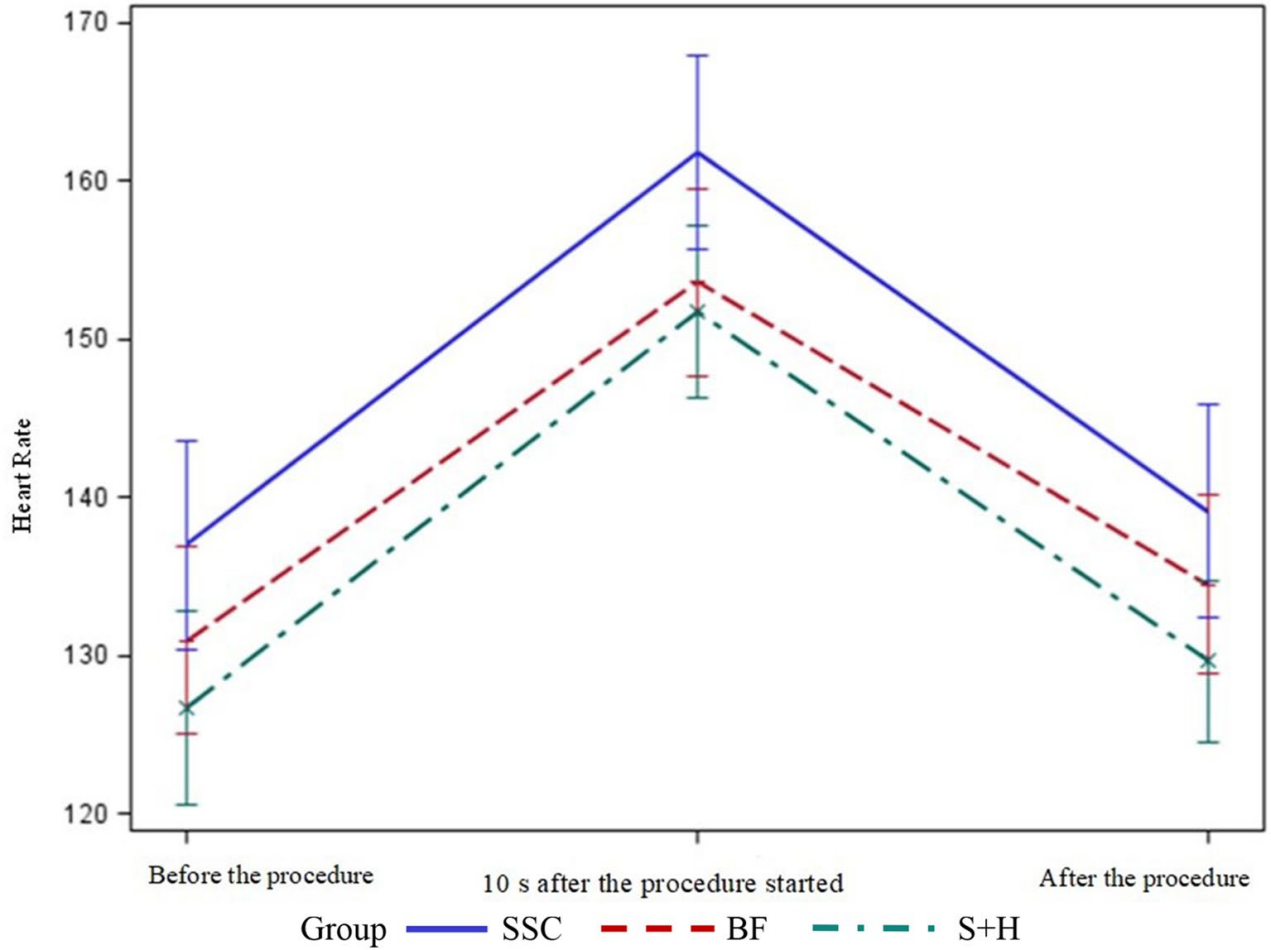
Distribution of Mean Heart Rate of Newborns by Group SSC: Skin-to-Skin Contact, BF: Breastfeeding, S + H: Swaddling + Holding

No significant difference was found between the groups’ mean heart rates before the procedure (*p* > 0.05); however, there was a statistically significant difference between their scores 10 s after the procedure started (*p* < 0.05) (Table 3; Fig. 3). In the pairwise comparison of the groups, the mean heart rates of the swaddling + holding and breastfeeding groups were significantly lower than that of the SSC group (*p* = 0.026 and *p* = 0.040, respectively).

**Table 3:** Distribution of measurements performed in newborns by group

Variable	Experimental groups										
	Breastfeeding^a^			SSC^b^			Swaddling + holding^c^			*X^2^	*p*
	M ± SD	Median	Min-Max	M ± SD	Median	Min-Max	M ± SD	Median	Min-Max		
**Heart rate**											
Before the procedure	130.97 **±** 15.88	133	91–161	137.00 **±** 17.79	138	103–167	126.73 **±** 16.33	123.5	100–173	5.178	0.075
10 s after the procedure started	153.60 **±** 15.81	151	128–197	161.80 **±** 16.43	160.5	130–193	151.73 **±** 14.65	152	122–176	6.155	**0.046** **a**,** c < b**
After the procedure	134.50 **±** 15.23	135	98–172	139.10 **±** 18.03	140.5	100–176	129.67 **±** 13.69	130.5	105–163	6.489	**0.038** **c < b**
**Oxygen saturation**											
Before the procedure	94.83 **±** 2.21	94.5	90–98	94.43 **±** 2.43	95	89–98	95.57 **±** 2.49	96	90–99	3.939	0.139
10 s after the procedure started	90.93 **±** 3.02	90	85–97	91.53 **±** 3.16	91	85–98	91.10 **±** 4.05	91	80–98	0.738	0.691
After the procedure	94.17 **±** 2.05	94.5	90–98	94.43 **±** 2.87	95	88–98	94.80 **±** 2.61	95	89–98	1.536	0.463
**Total crying time (sec)**	85.83 **±** 73.01	65	0-300	128.17 **±** 85.71	115	0-350	112.00 **±** 49.65	100	20–250	6.043	**0.048** **a < b**
**HL time (sec)**	132.00 ± 58.33	120	60–250	119.33 ± 40.42	105	60–240	118.67 ± 35.89	120	60–200	0.259	0.878
**Total time (sec)**	372.00 ± 58.33	360	300–490	359.33 ± 40.42	345	300–480	358.00 ± 36.62	360	300–440	0.256	0.879

**M**: Mean, **SD**: Standard Deviation **SSC**: skin-to-skin contact, *X^***2***^: Kruskal-Wallis Test

A statistically significant difference was found between the groups’ mean heart rates after the procedure (*p* < 0.05) (Table 3; Fig. 3). In the pairwise comparison of the groups, the mean heart rate of the swaddling + holding group was significantly lower than that of the SSC group (*p* = 0.033).

### Comparison of mean oxygen saturation and crying time scores of the groups

No statistically significant difference was found between the groups in terms of mean oxygen saturation scores before the procedure, 10 s after the procedure started, and after the procedure (*p* > 0.05) (Table 3).

There was a statistically significant difference between the mean total crying time of the groups (*p* < 0.05). In the pairwise comparison of the groups, the mean total crying time of the breastfeeding group was significantly lower than that of the SSC group (*p* = 0.021). Similarly, there was no statistically significant difference between the mean HL and total procedure times of the study groups (Table 3).

## Discussion

Previous studies have revealed that swaddling [3, 9], holding [6, 9, 11], swaddling + holding [12], breastfeeding [10, 11, 16–20], and SSC [5, 7, 10, 17] are effective methods to reduce the pain felt by newborns during the HL procedure. No studies comparing breastfeeding, swaddling + holding, and SSC during a HL were found in the literature. The results of the present study were discussed with those of studies comparing the three methods with a control group or using different nonpharmacologic methods.

In the present study, no statistically significant difference was found between the study groups’ (breastfeeding, SSC, and swaddling + holding) mean NIPS scores before the HL procedure. This result is similar to those of other studies in the literature [6, 8].

This study found a statistically significant difference between the mean NIPS scores of the study groups 10 s after the HL started. The mean NIPS score of the breastfeeding group at the 10th second after the procedure started was lower than that of the swaddling + holding group and the SSC group however, this difference between the breastfeeding and SSC groups was not significant. In the literature, it has been reported that breastfeeding during HL significantly reduces the level of pain in studies where it is used alone [19] or in combination with several non-pharmacological methods [6, 12]. According to a Cochrane systematic review, breastfeeding reduces the mean NIPS score compared to maternal holding, swaddling, and swaddling + holding [28]. Direct breast-feeding was stated as the best method of non-pharmacological pain management compared to all other (holding, skin-to-skin contact, topical anesthetics, and music), and was preferable even to administration of glucose/sucrose in full-term infants [29]. In this context, breastfeeding can effectively manage pain during invasive interventions such as HL procedures in newborns due to its easy application, cost, and lack of negative effects, as well as its ability to stop pain by stimulating the release of opioids and blocking pain fibers [30].

The present study found a statistically significant difference between the study groups’ mean NIPS scores after the HL procedure. The breastfeeding group’s mean NIPS score after the procedure was lower than those of the SSC and the swaddling + holding groups. This research result is similar to theliterature [10]. Breastfeeding is more effective than SSC and swaddling + holding in controlling pain immediately after the HL procedure. The pain level decreases when SSC and swaddling + holding methods are used together with effective non-pharmacological methods (such as breastfeeding or breast milk) immediately after the HL procedure. However, non-pharmacological methods are used in very few health institutions in Turkey during the HL procedure.

Heart rate and blood pressure increase during pain in neonates [1]. The present study found no statistically significant difference between the groups’ mean heart rates before the HL procedure. The result of our study is similar to the study of Obeidat and Shuriquie [6]; however, it differs from other two studies [3, 5] because they only examined the effect of one nonpharmacologic method on pain level before the HL.

In this study, it was found that the practices that prevented the increase in heart rate during HL were breastfeeding and swaddling + holding. A cochrane systematic review reported that breastfeeding reduces the increase in heart rate compared to maternal holding, SSC, and bottle feeding [28]. In this context, it would be useful to use SSC together with breastfeeding and similar methods to reduce the pain and heart rate felt by newborns during the HL procedure.

In the study, the mean heart rate of the swaddling + holding group was significantly lower than that of the SSC group after the HL procedure. Previous studies reported that the mean heart rate scores of the swaddling [3], kangaroo care (1st min and 2nd min after the procedure) [8], and SSC (5th min after the procedure) [5] groups after an HL procedure were lower than that of the control group. Swaddling + holding, can be easily used by nurses to reduce pain and thus heart rate after the HL procedure.

When the newborn is exposed to painful procedures, respiration is rapid and superficial, and oxygen saturation decreases [1]. The present study found no statistically significant difference between the mean oxygen saturation of the study groups at the measurement times.

In the literature, one study reported no difference between mean oxygen saturation of breastfeeding + maternal holding and maternal holding groups before the HL procedure [6]. In other studies, it has been reported that there is no difference in oxygen saturation between the groups in which different non-pharmacological methods and the control group before [3, 5, 8], during [3, 8] and after [5, 8] the HL procedure. It is thought that, in the present study, the mean oxygen saturation scores were similar to each other because the three different methods used during the HL procedure played a role in reducing the pain felt by newborns. The fact that the oxygen saturation scores of the groups were similar to each other is thought to be due to the effects of the three different methods used in this study to reduce pain during the HL procedure.

Newborns react verbally by crying besides physiological and behavioral changes during a painful intervention [31]. There was a significant difference between the mean total crying time of the groups in the present study. The mean total crying time of the breastfeeding group was significantly lower than that of the SSC group. A Cochrane systematic review reported that breastfeeding reduced crying time compared to no intervention, lying on the table, maternal holding, SSC, and bottle feeding [28]. Karapınar [10] reported that the crying time of the breastfeeding group was lower than the SSC group. In the study conducted by Okan et al. [7], the crying time of the SSC + breastfeeding group was shorter than that of the SSC group. The study by Yılmaz and İnal [12] showed that the total crying time was lower in the swaddling + holding + breastfeeding group than in the swaddling + holding group. In the present study, it is thought that the crying time of the breastfeeding group was shorter than the swaddling + holding and SSC groups due to the calming effect of breastfeeding and breast milk. To soothe newborns, we suggest breastfeeding + swaddling + holding or breastfeeding + SSC during a HL.

In this study, the mean HL time and total procedure time of the newborns in the study groups were similar. Chang et al. [17] reported that there was a difference between the control group and four intervention groups (oral sucrose, breastfeeding, non-nutritive sucking, and SSC) in terms of the HL procedure time; however, there was no difference between the groups in the comparison of the intervention groups with one another. This result is similar to that of the present study.

The results obtained from the present analysis can be extended to other procedures performed in neonatal care. Clinical outcomes of diseases should be evaluated with appropriate scales, especially in cases with increased sensitivity and high risk of pain (due to increased painful sensitivity or surgical interventions and tissue damage), such as congenital skin defects [32], genetic diseases [33] or malformations [34].

### Limitations

This study has some limitations. These were blinding was not used, the study was limited in time because it was derived from a master’s thesis, there was no quiet room reserved for the HL procedure, and study data were collected from a total of 90 newborns, 30 in each group, due to the COVID-19 pandemic.

The wardroom was for two people and the beds were separated with a curtain to ensure privacy, which made it difficult to maintain silence while recording the newborn’s cries during the HL.

## Conclusions

As a result, direct breastfeeding is the most effective method compared to swaddling + holding method in reducing pain the newborns feel during the HL procedure. Direct breastfeeding is the most effective method to reducing the pain newborns feel and to calm them down after the HL procedure. Breastfeeding and swaddling + holding are more effective than SSC in reducing heart rate during the HL. Breastfeeding is more effective than SSC in reducing total crying time.

New data should be added to the body of knowledge in order to reach a consensus on nonpharmacological methods to be used in pain management in newborns. In this context, it is recommended to conduct more comprehensive studies.

## Data Availability

The datasets used and/or analysed during the current study are available from the corresponding author on reasonable request.

## Abbreviations

NIPS: Neonatal Infant Pain Scale
SSC: L Skin-to-Skin Contact
HL: Heel Lance
BF: Breastfeeding
